# Supplementary material for: NAMPT-derived NAD+ fuels PARP1 to promote skin inflammation through parthanatos cell death
Source: PLoS Biol. 2021 Nov 8;19(11):e3001455. doi: 10.1371/journal.pbio.3001455 (PMC8601609; doi:10.1371/journal.pbio.3001455)
Supplement: S1 Fig — Genetic and pharmacological inhibition of Nampt alleviates skin inflammation and restores epithelial integrity in Spint1a-deficient larvae. (A) Neutrophil distribution of wild-type and Spint1a-deficient larvae treated with the pharmacological inhibitors of Nampt GMX1778 and FK-866. (B) Representative merge images (brightfield and red channels) of lyz:dsRED zebrafish larvae of every group are shown. (C) For genetic inhibition using CRISPR/Cas-9 technology, 1-cell stage zebrafish eggs were microinjected and imaging was performed in 3 dpf larvae (D). Quantification of the percentage of neutrophils out of the CHT in Spint1a-deficient larvae upon knockdown of Nampta/Namptb. (E) Representative merge images (brightfield and red channel) of lyz:dsRED zebrafish larvae of every group are shown (F) Analysis of genome editing efficiency in larvae injected with control or nampta and namptb crRNA/Cas-9 complexes and quantification rate of NHEJ-mediated repair showing all INDELs (https://tide.nki.nl/). Each dot represents one individual, and the mean ± SEM for each group is also shown. p-Values were calculated using 1-way ANOVA and Tukey multiple range test and t test. ***p ≤ 0.001, ****p ≤ 0.0001. (G) Inflammation and oxidative stress determination in zebrafish larvae [21]. Inflammation was scored by using 2 different approaches: (i) the lyz:dsRED zebrafish transgenic line was used to quantify the percentage of neutrophils out of the CHT, i.e., neutrophil dispersion; and (ii) the nfkb:eGFP zebrafish transgenic line was used to determine NFKB activity by quantification of fluorescence intensity in the drawn white box. For oxidative stress, analysis of fluorescence intensity of the ROI (white box) of larvae preloaded with an H2O2 fluorogenic probe. The data underlying this figure can be found in S1 Data. ANOVA, analysis of variance; CHT, caudal hematopoietic tissue; INDEL, insertion and deletion; Nampt, nicotinamide phosphoribosyltransferase; NHEJ, nonhomologous end joining; ROI, r [file pbio.3001455.s001.pdf]

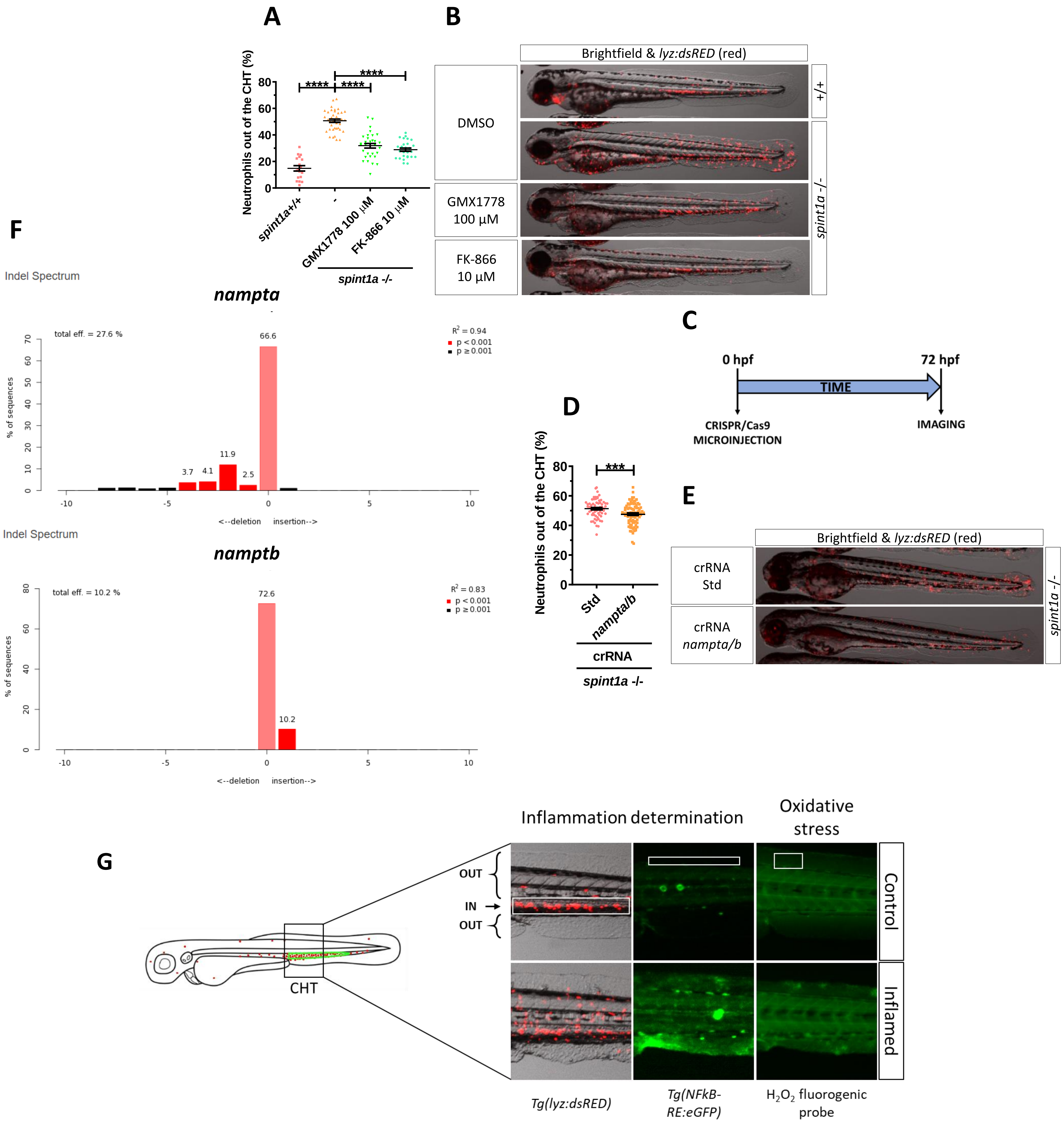

S1 Figure, related to Figure 2. Genetic and pharmacological inhibition of Nampt alleviates skin inflammation and restores epithelial integrity in *spint1a* –deficient larvae. (A) Neutrophil distribution of wild type and Spint1a-deficient larvae treated with the pharmacological inhibitors of Nampt GMX1778 and FK-866. (B) Representative merge images (brightfield and red channels) of *lyz:dsRED* zebrafish larvae of every group are shown. (C) For genetic inhibition using CRISPR/Cas9 technology, one-cell stage zebrafish eggs were microinjected and imaging was performed in 3 dpf larvae (D). Quantification of the percentage of neutrophils out of the CHT in Spint1a-deficient larvae upon knockdown of Nampta/Namptb. (E) Representative merge images (brightfield and red channel) of *lyz:dsRED* zebrafish larvae of every group are shown (F) Analysis of genome editing efficiency in larvae injected with control or *nampta* and *namptb* crRNA/Cas9 complexes and quantification rate of non-homologous end-joining mediated repair (NHEJ) showing all insertions and deletions (INDELs) (https://tide.nki.nl/). Each dot represents one individual and the mean ± S.E.M. for each group is also shown. P values were calculated using one-way ANOVA and Tukey multiple range test and t-Test. ***p≤0.001, ****p≤0.0001. (G) Inflammation and oxidative stress determination in zebrafish larvae (Candel et al., 2014). Inflammation was scored by using two different approaches: i) the *lyz:dsRED* zebrafish transgenic line was used to quantify the percentage of neutrophils out of the CHT, i.e. neutrophil dispersion and ii) the *nfkb:eGFP* zebrafish transgenic line was used to determine NFKB activity by quantification of fluorescence intensity in the drawn white box. For oxidative stress, analysis of fluorescence intensity of the region of interest (white box) of larvae preloaded with an H_2O_2 fluorogenic probe. The data underlying this figure can be found in S1 Data.
