# Supplementary material for: NAMPT-derived NAD+ fuels PARP1 to promote skin inflammation through parthanatos cell death
Source: PLoS Biol. 2021 Nov 8;19(11):e3001455. doi: 10.1371/journal.pbio.3001455 (PMC8601609; doi:10.1371/journal.pbio.3001455)
Supplement: S2 Fig — ROS scavenging and inhibition of NADPH oxidases rescue skin neutrophil recruitment and skin morphological alterations of Spint1a-deficient larvae. Quantification of keratinocyte aggregation foci in the tail of lyz:dsRED larvae shown in Fig 3(A, C, E, G, I) and detailed representative merge images (brightfield and red channel) (B, D, F, H, J) upon their treatment with with vehicle (DMSO), 100 μM NAC (A, B), 100 μM mito-TEMPO (MT), and 100 nM tempol (T) (C, D), 250 μM apocynin (E, F), or upon genetic inhibition of nox1 and nox5 (G, H), and nox4 (I, J). White arrows indicate keratinocyte aggregates. Each dot represents one individual, and the mean ± SEM for each group is also shown. p-Values were calculated using 1-way ANOVA and Tukey multiple range test and t test. ****p ≤ 0.0001. The data underlying this figure can be found in S1 Data. ANOVA, analysis of variance; NAC, N-acetylcysteine; ROS, reactive oxygen species. (PDF) [file pbio.3001455.s002.pdf]

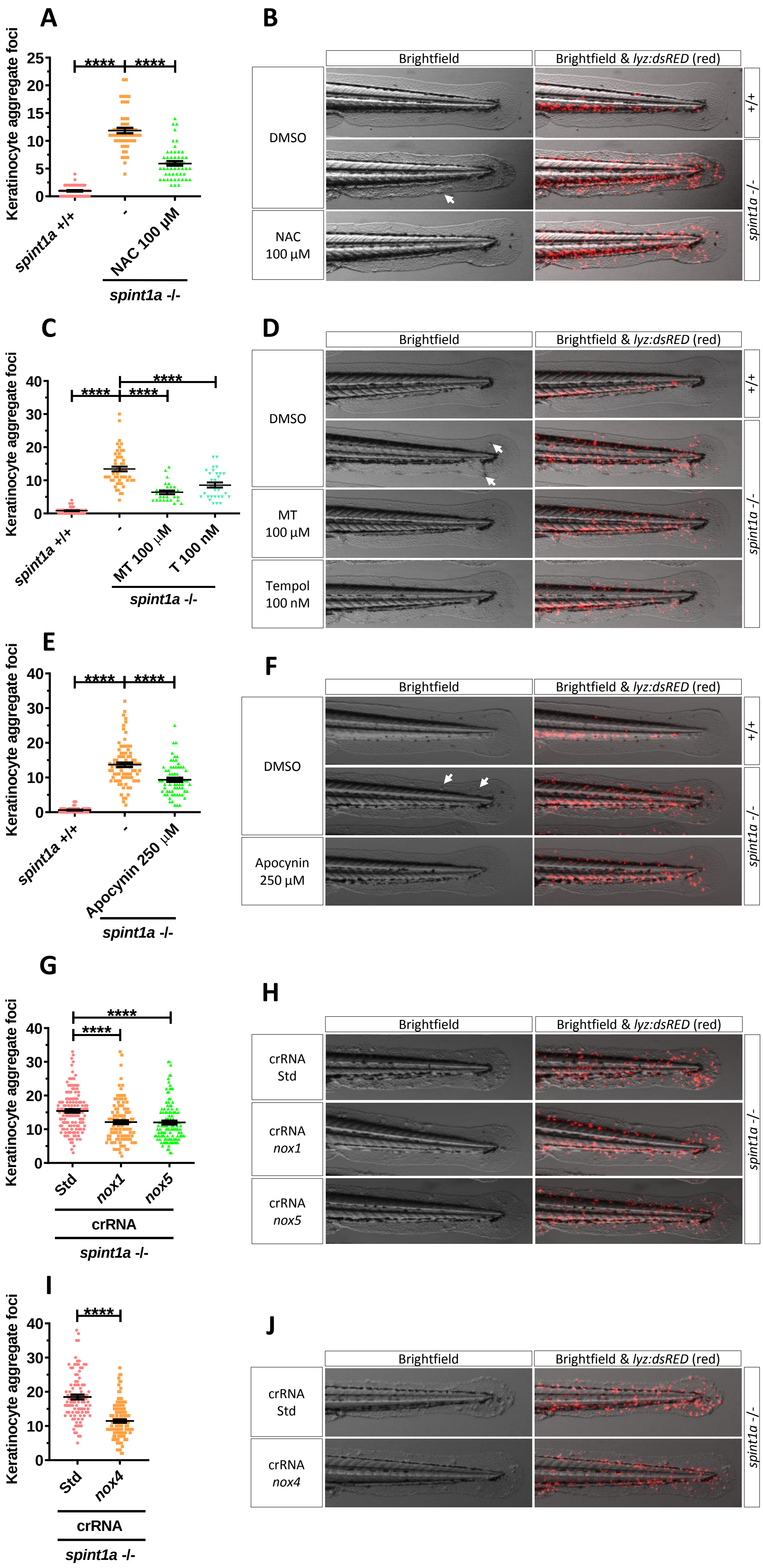

S2 Figure, related to Figure 3. ROS scavenging and inhibition of NADPH-oxidases rescue skin neutrophil recruitment and skin morphological alterations of Spint1-deficient larvae. Quantification of keratinocyte aggregation foci in the tail of *lyz:dsRED* larvae shown in Figure 3 (A, C, E, G, I) and detailed representative merge images (brightfield and red channel) (B, D, F, H, J) upon their treatment with with vehicle (DMSO), 100 µM N-acetylcysteine (NAC) (A, B), 100 µM mito-TEMPO (MT) and 100 nM tempol (T) (C, D), 250 µM apocynin (E, F), or upon genetic inhibition of *nox1* and *nox5* (G, H), and nox4 (I, J). White arrows indicate keratinocyte aggregates. Each dot represents one individual and the mean ± S.E.M. for each group is also shown. P values were calculated using one-way ANOVA and Tukey multiple range test and t-Test. ****p≤0.0001. The data underlying this figure can be found in S1 Data.
