# Supplementary material for: NAMPT-derived NAD+ fuels PARP1 to promote skin inflammation through parthanatos cell death
Source: PLoS Biol. 2021 Nov 8;19(11):e3001455. doi: 10.1371/journal.pbio.3001455 (PMC8601609; doi:10.1371/journal.pbio.3001455)
Supplement: S3 Fig — Efficiency of crRNA for nox1, nox4, and nox5. Analysis of genome editing efficiency in larvae injected with control or nox1 (A), nox4 (B), and nox5 (C) crRNA/Cas-9 complexes and quantification rate of NHEJ-mediated repair showing all INDELs (https://tide.nki.nl/). INDEL, insertion and deletion; NHEJ, nonhomologous end joining. (PDF) [file pbio.3001455.s003.pdf]

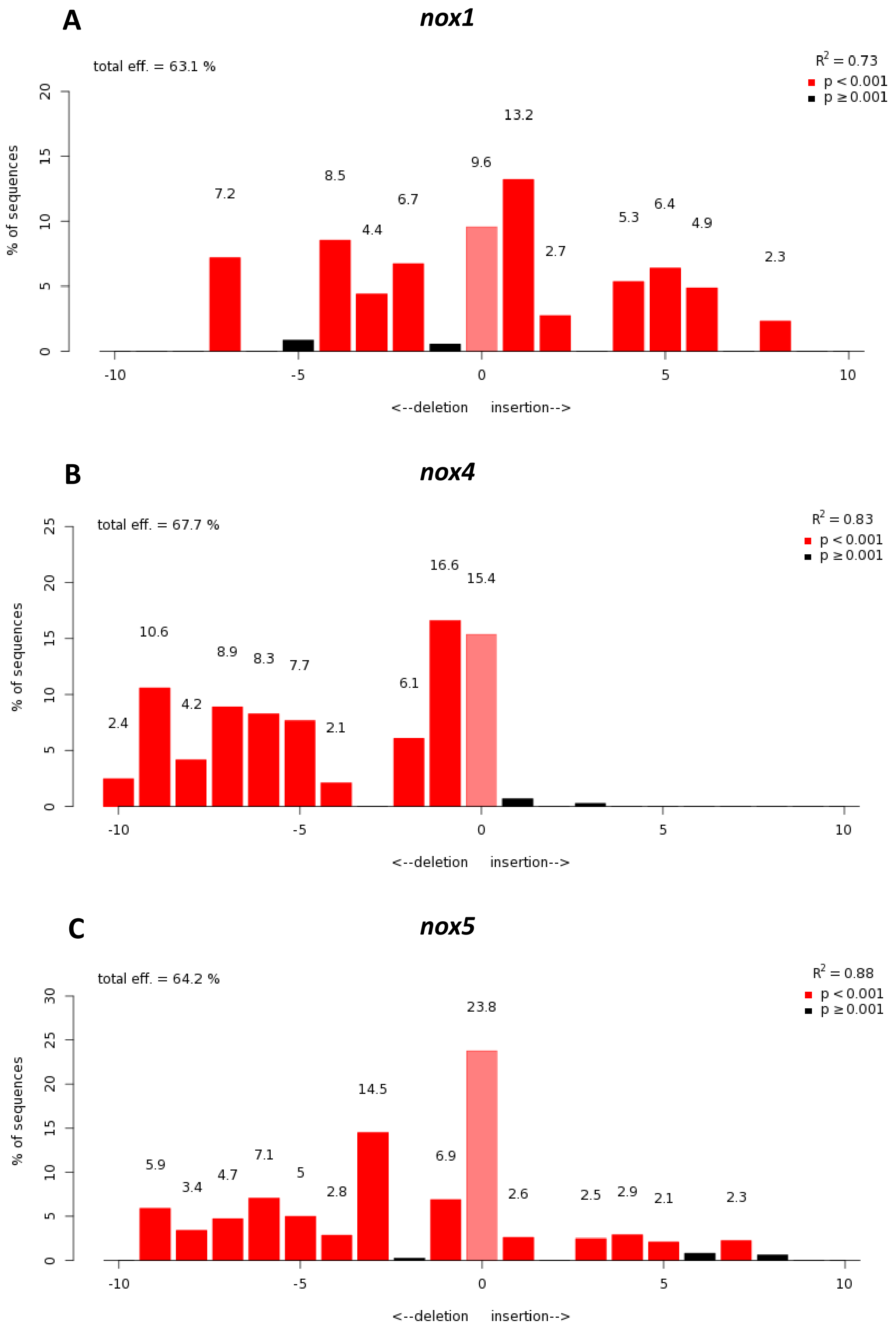

S3 Figure, related to Figure 3. Efficiency of crRNA for *nox1*, *nox4* and *nox5*. Analysis of genome editing efficiency in larvae injected with control or *nox1* (A), *nox4* (B) and *nox5* (C) crRNA/Cas9 complexes and quantification rate of non-homologous end-joining mediated repair (NHEJ) showing all insertions and deletions (INDELs) (https://tide.nki.nl/).
