# Supplementary material for: NAMPT-derived NAD+ fuels PARP1 to promote skin inflammation through parthanatos cell death
Source: PLoS Biol. 2021 Nov 8;19(11):e3001455. doi: 10.1371/journal.pbio.3001455 (PMC8601609; doi:10.1371/journal.pbio.3001455)
Supplement: S4 Fig — Genetic and pharmacological inhibition of Nampt and Parp1 diminishes PARylation, skin inflammation, and restores epithelial integrity in Spint1a-deficient larvae. (A, B) Quantification of keratinocyte aggregates and detailed representative merge images (brightfield and red channels) of wild-type and Spint1a-deficient zebrafish treated with vehicle (DMSO) or 100 μM olaparib shown in Fig 4A. (C–E) Neutrophil distribution (C) and keratinocyte aggregates (D) of Spint1a-deficient larvae injected with control or parp1 crRNA/Cas-9 complexes. Representative images are shown in E. (F) Analysis of genome editing efficiency in larvae injected with control or parp1 crRNA/Cas-9 complexes and quantification rate of NHEJ-mediated repair showing all INDELs (https://tide.nki.nl/). (G, H) Western blots with anti-PAR and anti-β-actin of tail fold (red boxed area) lysates from 3 dpf wild-type and Spint1a-deficient zebrafish larvae treated for 48 hours with 10 μM FK-866 or 100 μM olaparib. The relative abundance of PAR with respect to β-actin is shown in each lane. Each dot represents one individual, and the mean ± SEM for each group is also shown. p-Values were calculated using 1-way ANOVA and Tukey multiple range test and t test. ****p ≤ 0.0001 (C). Representative merge images (brightfield and red channel) of lyz:dsRED zebrafish larvae of every group are shown (D). The data underlying this figure can be found in S1 Data. ANOVA, analysis of variance; INDEL, insertion and deletion; NHEJ, nonhomologous end joining; Nampt, nicotinamide phosphoribosyltransferase; PAR, poly(ADP-ribose). (PDF) [file pbio.3001455.s004.pdf]

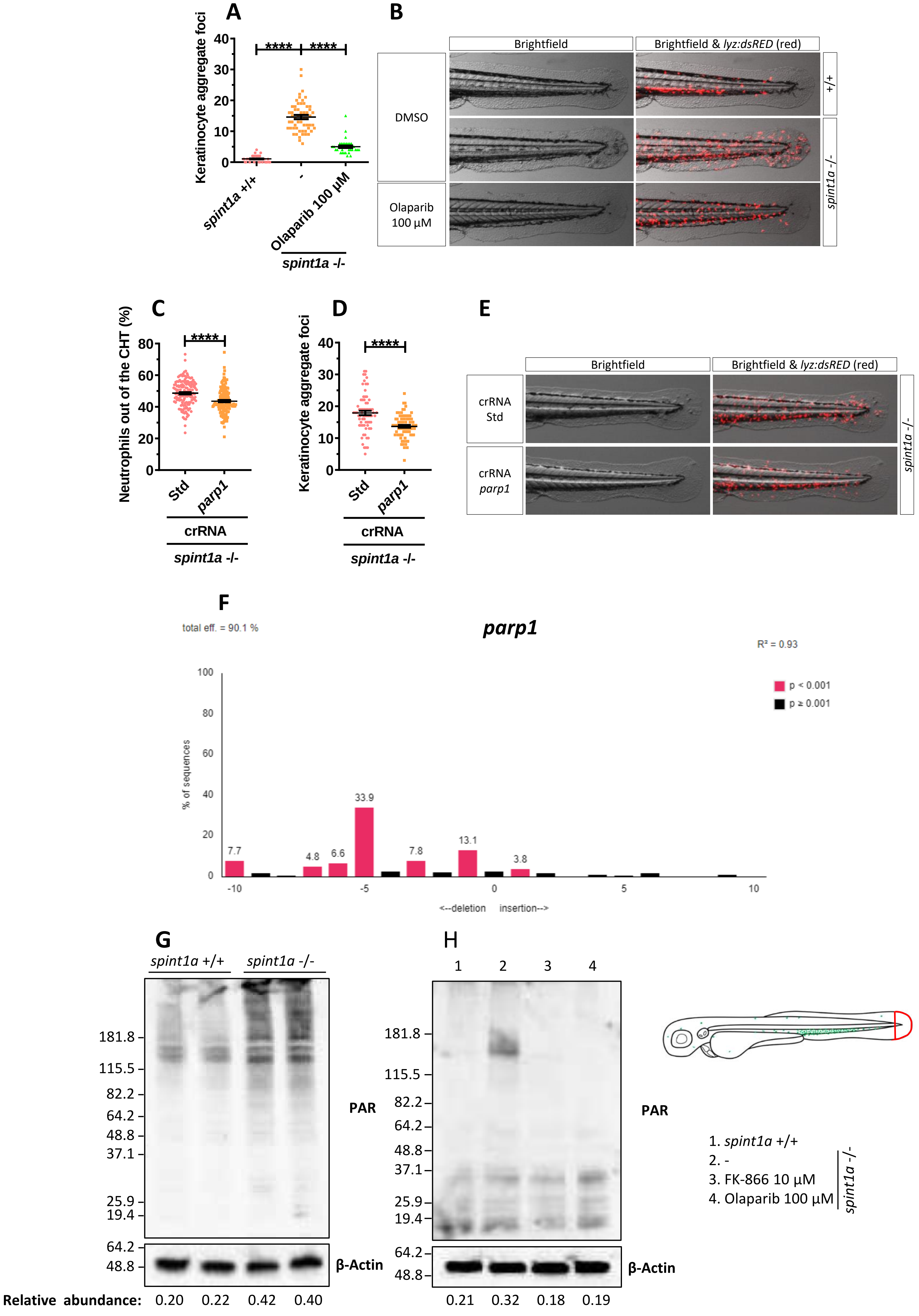

S4 Figure, related to Figure 4. Genetic and pharmacological inhibition of Nampt and Parp1 diminishes PARylation, skin inflammation and restores epithelial integrity in Spint1a-deficient larvae. (A, B) Quantification of keratinocyte aggregates and detailed representative merge images (brightfield and red channels) of wild type and Spint1a-deficient zebrafish treated with vehicle (DMSO) or 100 µM olaparib shown in Figure 4A. (C-E) Neutrophil distribution (C) and keratinocyte aggregates (D) of Spint1a-deficient larvae injected with control or *parp1* crRNA/Cas9 complexes. Representative images are shown in E. (F) Analysis of genome editing efficiency in larvae injected with control or *parp1* crRNA/Cas9 complexes and quantification rate of non-homologous end-joining mediated repair (NHEJ) showing all insertions and deletions (INDELs) (https://tide.nki.nl/). (G, H) Western blots with anti-PAR and anti-β-actin of tail fold (red boxed area) lysates from 3 dpf wild type and Spint1a-deficient zebrafish larvae treated for 48 hours with 10 µM FK-866 or 100 µM olaparib. The relative abundance of PAR with respect to β-actin is shown in each lane. Each dot represents one individual and the mean ± S.E.M. for each group is also shown. P values were calculated using one-way ANOVA and Tukey multiple range test and t-Test. ****p≤0.0001 (C). Representative merge images (brightfield and red channel) of *lyz:dsRED* zebrafish larvae of every group are shown (D). The data underlying this figure can be found in S1 Data.
