# Supplementary material for: NAMPT-derived NAD+ fuels PARP1 to promote skin inflammation through parthanatos cell death
Source: PLoS Biol. 2021 Nov 8;19(11):e3001455. doi: 10.1371/journal.pbio.3001455 (PMC8601609; doi:10.1371/journal.pbio.3001455)
Supplement: S5 Fig — FK-866 and olaparib improve skin epithelial integrity in psoriasis mutants. (A, B) Determination of the skin phenotype of 2.5 dpf zebrafish Atp1b1a-deficient larvae treated 1.5 days with 50 μM FK-866 or 100 μM olaparib. (C) Representative bright field images of zebrafish larvae of every group are shown. p-Values were calculated using chi-squared and Fisher exact test *p ≤ 0.05, ****p ≤ 0.0001. The data underlying this figure can be found in S1 Data. (PDF) [file pbio.3001455.s005.pdf]

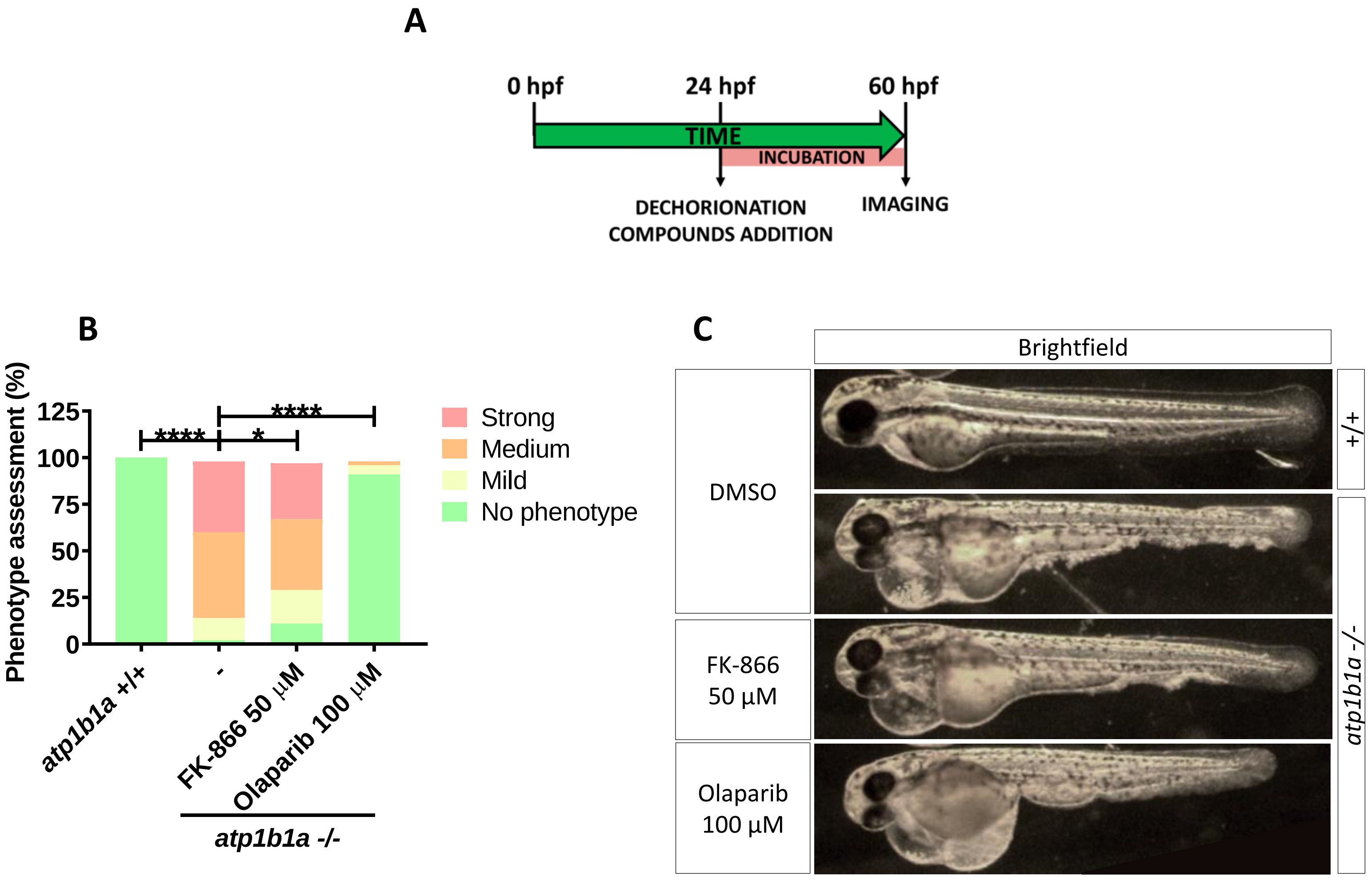

S5 Figure, related to Figures 2 and 4. FK-866 and olaparib improve skin epithelial integrity in *psoriasis* mutants. (A, B) Determination of the skin phenotype of 2.5 dpf zebrafish Atp1b1a-deficient larvae treated 1.5 days with 50 µM FK-866 or 100 µM olaparib. (C) Representative bright field images of zebrafish larvae of every group are shown. P values were calculated using Chi-square and Fisher´s exact test *p≤0.05, ****p≤0.0001. The data underlying this figure can be found in S1 Data.
