# Supplementary material for: NAMPT-derived NAD+ fuels PARP1 to promote skin inflammation through parthanatos cell death
Source: PLoS Biol. 2021 Nov 8;19(11):e3001455. doi: 10.1371/journal.pbio.3001455 (PMC8601609; doi:10.1371/journal.pbio.3001455)
Supplement: S6 Fig — Inhibition of parthanatos rescues morphological skin alterations of Spint1a-deficient larvae. Quantification of keratinocyte aggregates (A, E, H) and detailed representative merge images (brightfield and red channels) (B, F, I) of wild-type and Spint1a-deficient larvae treated with vehicle (DMSO) or 10 nM NP (A, B, C, D), aifm1 genetic inhibition (E, F) and parga mRNA overexpression (H, I) of zebrafish larvae shown in Fig 5. (C) Quantification of the percentage of nuclear Aifm1 positive cells (white arrows) in zebrafish skin, calculated as the ratio between the number of keratinocytes in which Aifm1 is found in the nucleus and total keratinocyte number analyzed. (D) Laser confocal microscopy Z stack of Aifm1 immunostaining (red) in 72 hpf wild-type and Spint1a-deficient larvae treated for 48 hours with 10 nM NP. Samples were counterstained with DAPI (blue). Normal keratinocytes, keratinocyte aggregates, and neuromast are shown. (G) Analysis of genome editing efficiency of larvae injected with control or aifm1 crRNA/Cas-9 complexes and quantification rate of NHEJ-mediated repair showing all INDELs (https://tide.nki.nl/). Each dot represents one individual, and the mean ± SEM for each group is also shown. p-Values were calculated using 1-way ANOVA and Tukey multiple range test and t test. ***p ≤ 0.001, ****p ≤ 0.0001. The data underlying this figure can be found in S1 Data. ANOVA, analysis of variance; INDEL, insertion and deletion; NHEJ, nonhomologous end joining; NP, N-phenylmaleimide. (PDF) [file pbio.3001455.s006.pdf]

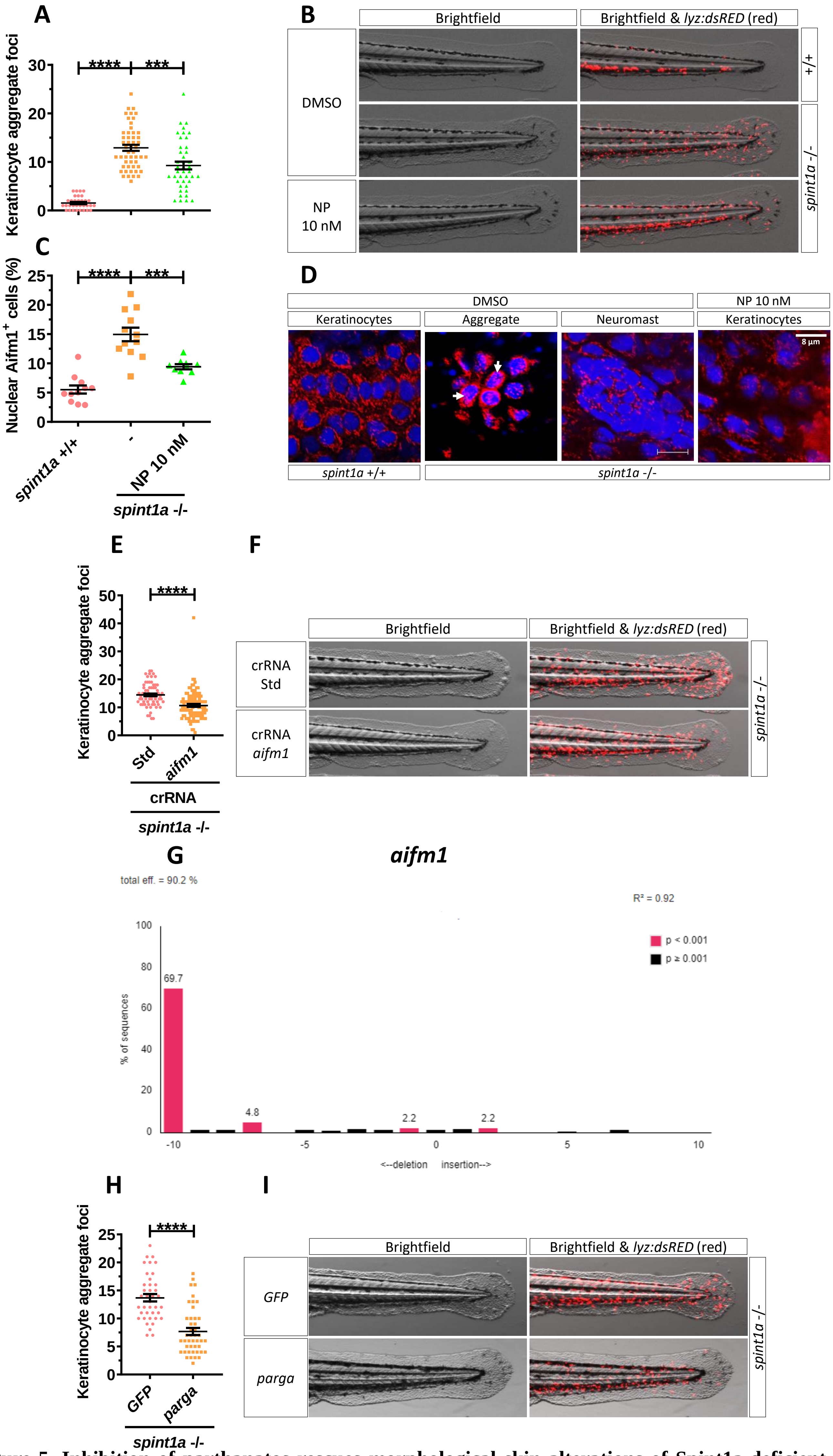

S6 Figure, related to Figure 5. Inhibition of parthanatos rescues morphological skin alterations of Spint1a-deficient larvae. Quantification of keratinocyte aggregates (A, E, H) and detailed representative merge images (brightfield and red channels) (B, F, I) of wild type and Spint1a-deficient larvae treated with vehicle (DMSO) or 10 nM N-phenylmaleimide (NP) (A, B, C, D), *aifm1* genetic inhibition (E, F) and *parga* mRNA overexpression (H, I) of zebrafish larvae shown in Figure 5. (C) Quantification of the percentage of nuclear Aifm1 positive cells (white arrows) in zebrafish skin, calculated as the ratio between the number of keratinocytes in which Aifm1 is found in the nucleus and total keratinocyte number analyzed. (D) Laser confocal microscopy Z stack of Aifm1 immunostaining (red) in 72 hpf wild type and Spint1a-deficient larvae treated for 48 hours with 10 nM NP. Samples were counterstained with DAPI (blue). Normal keratinocytes, keratinocyte aggregates and neuromast are shown. (G) Analysis of genome editing efficiency of larvae injected with control or *aifm1* crRNA/Cas9 complexes and quantification rate of non-homologous end-joining mediated repair (NHEJ) showing all insertions and deletions (INDELs) (https://tide.nki.nl/). Each dot represents one individual and the mean ± S.E.M. for each group is also shown. P values were calculated using one-way ANOVA and Tukey multiple range test and t-Test. ***p≤0.001, ****p≤0.0001. The data underlying this figure can be found in S1 Data.
