# Supplementary material for: NAMPT-derived NAD+ fuels PARP1 to promote skin inflammation through parthanatos cell death
Source: PLoS Biol. 2021 Nov 8;19(11):e3001455. doi: 10.1371/journal.pbio.3001455 (PMC8601609; doi:10.1371/journal.pbio.3001455)
Supplement: S7 Fig — NAD+ and PAR metabolic pathways. NAM, NMN, and NAD+ can be taken up by specific transporters. NAD+ biosynthetic pathways generate NAD+ from different precursors, de novo pathway employs dietary Trp or alternatively quinolinic acid (QA), NAD+ Salvage pathway mainly uses NAM but NMN and NR can also act as precursors. However, Preiss–Handler pathway utilizes NA. NAD+ is consumed by CD38 yielding NAM and ADPR or cADPR. NNMT also reduces NAD+ pool mediating the reaction between NAM and SAM to produce N-methylnicotinamide (1-MNA) and SAH. Finally, PARP1 synthesizes PAR by using NAD+ as a cofactor. PAR is degraded to ADPR mediated by different PAR hydrolases which cleave specific chemical linkages (exo- or endoglycosidically). Metabolic intermediates: NFK, NAAD, and NAMN. NAD+ transporter: CX43. ADPR, adenosine diphosphoribose; caDPR, cyclic ADPR; CX43, connexin 43; NA, nicotinic acid; NAAD, nicotinic acid adenine dinucleotide; NAD+, nicotinamide adenine dinucleotide; NAM, nicotinamide; NAMN, nicotinic acid mononucleotide; NFK, N-formylkynurenine; NMN, nicotinamide mononucleotide; NR, nicotinamide riboside; PAR, poly(ADP-ribose); SAH, S-adenosylhomocysteine; SAM, S-adenosylmethionine; Trp, tryptophan. (PDF) [file pbio.3001455.s007.pdf]

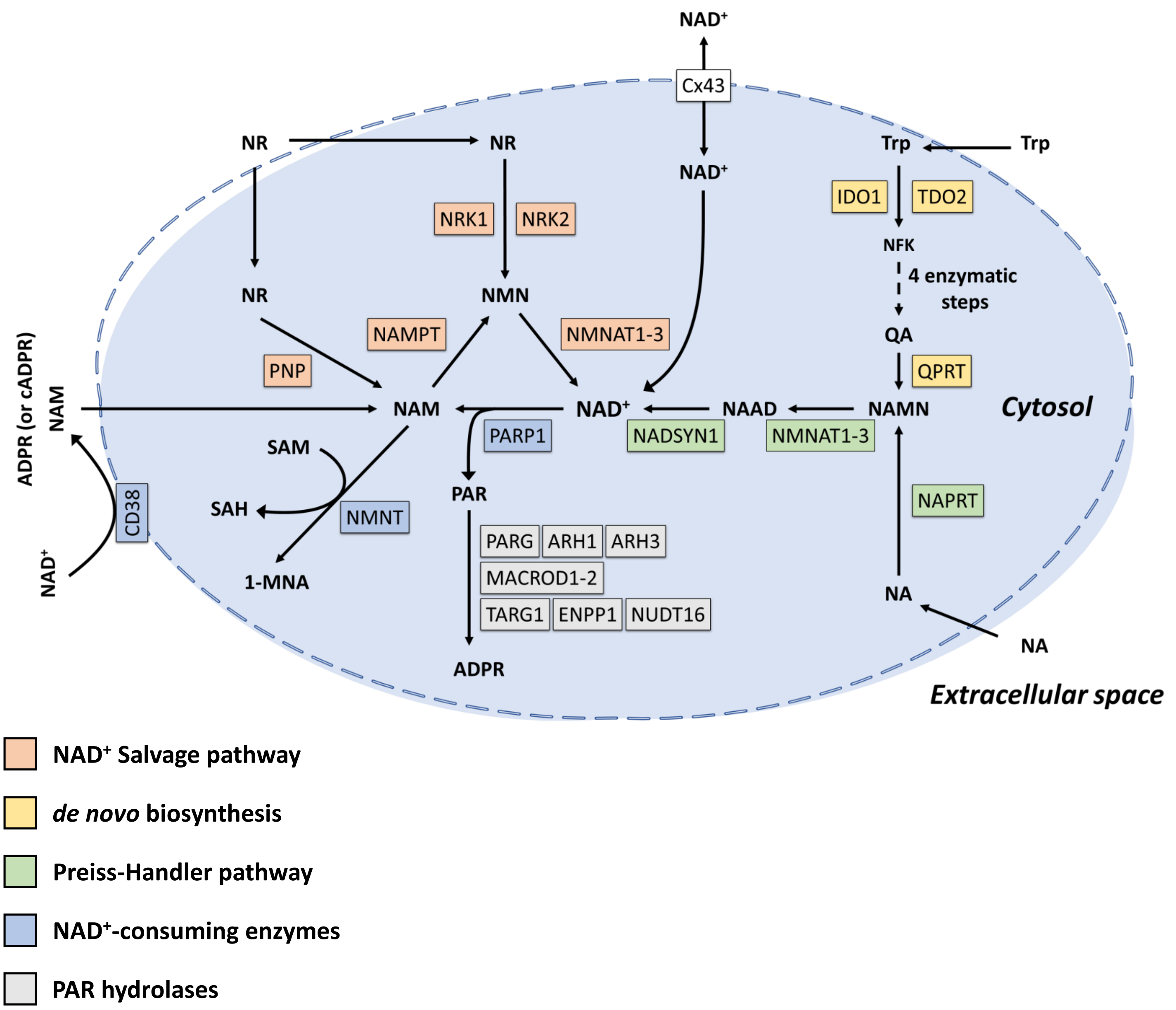

S7 Figure, related to Figure 6. NAD$^+$ and PAR metabolic pathways. NAM, NMN and NAD$^+$ can be taken up by specific transporters . NAD$^+$ biosynthetic pathways generate NAD$^+$ from different precursors, *de novo* pathway employs dietary tryptophan (Trp) or alternatively quinolinic acid (QA), NAD$^+$ Salvage pathway mainly uses nicotinamide (NAM) but nicotinamide mononucleotide (NMN) and nicotinamide riboside (NR) can also act as precursors. However, Preiss-Handler pathway utilizes nicotinic acid (NA). NAD$^+$ is consumed by CD38 yielding NAM and adenosine diphosphoribose (ADPR) or cyclic ADPR (cADPR). NNMT also reduces NAD$^+$ pool mediating the reaction between NAM and S-adenosylmethionine (SAM) to produce N-methylnicotinamide (1-MNA) and S-adenosylhomocysteine (SAH). Finally, PARP1 synthesizes PAR by using NAD$^+$ as a cofactor. PAR is degraded to ADPR mediated by different PAR hydrolases which cleave specific chemical linkages (exo- or endoglycosidically). Metabolic intermediates: N-formylkynurenine (NFK), nicotinic acid adenine dinucleotide (NAAD) and nicotinic acid mononucleotide (NAMN). NAD$^+$ transporter: connexin 43 (CX43).
