# Supplementary material for: NAMPT-derived NAD+ fuels PARP1 to promote skin inflammation through parthanatos cell death
Source: PLoS Biol. 2021 Nov 8;19(11):e3001455. doi: 10.1371/journal.pbio.3001455 (PMC8601609; doi:10.1371/journal.pbio.3001455)
Supplement: S9 Fig — Differential expression profiles of genes encoding parthanatos components in psoriasis. Transcriptomic data from human psoriasis (GDS4602) samples from the GEO database. Nonlesional and lesional psoriasis skin were compared with healthy skin samples. Each dot represents one individual, and the mean ± SEM for each group is also shown. p-Values were calculated using 1-way ANOVA and Tukey multiple range test (A) and t test (B). ns, not significant. *p ≤ 0.05, **p ≤ 0.01, ***p ≤ 0.001, ****p ≤ 0.0001. The data underlying this figure can be found in S1 Data. ANOVA, analysis of variance; GEO, Gene Expression Omnibus. (PDF) [file pbio.3001455.s009.pdf]

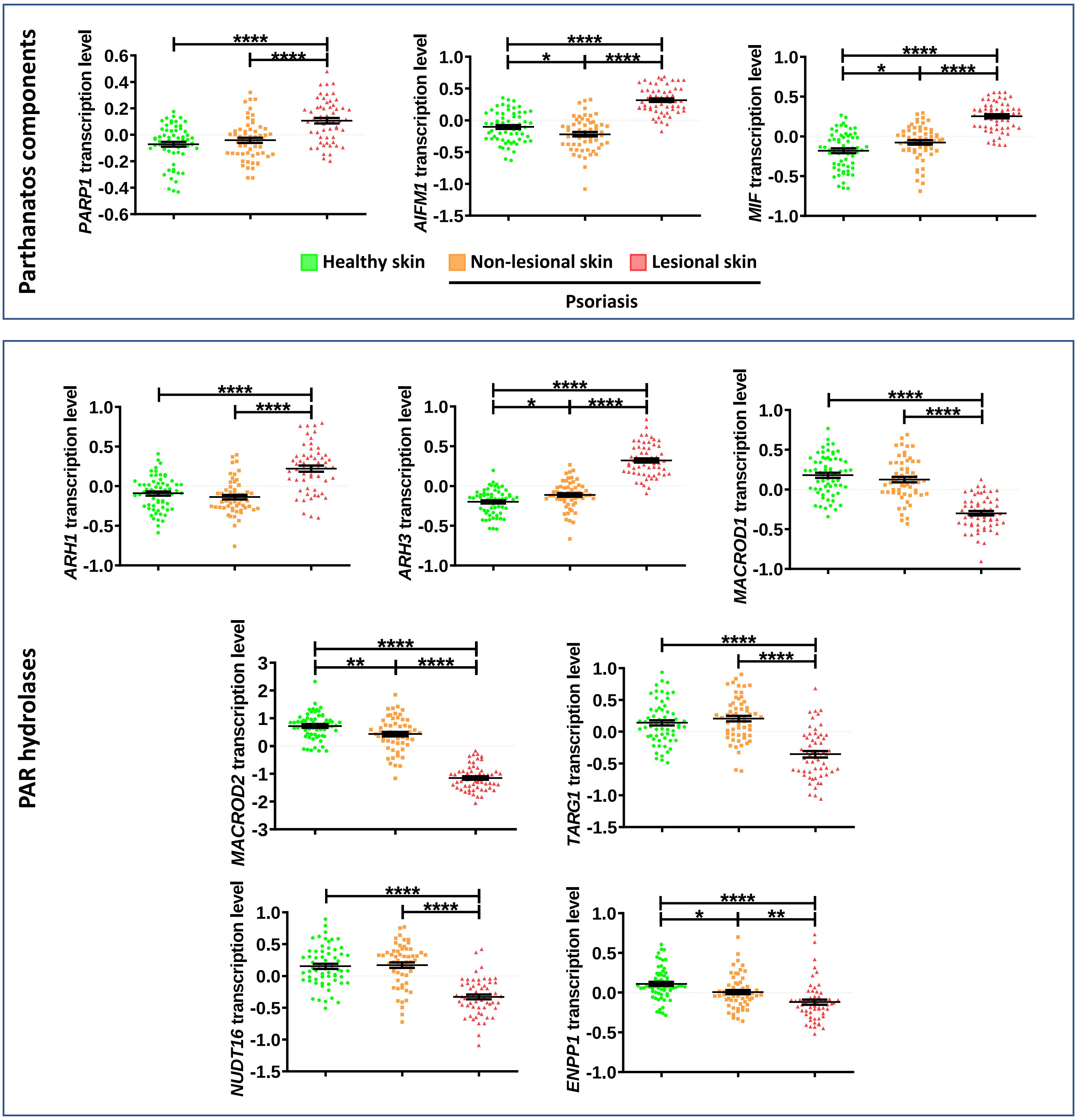

S9 Figure, related to Figure 6. Differential expression profiles of genes encoding parthanatos components in psoriasis and atopic dermatitis. Transcriptomic data from human psoriasis (GDS4602) samples from the Gene Expression *Omnibus* (GEO) database. Non-lesional and lesional psoriasis skin were compared with healthy skin samples. Each dot represents one individual and the mean ± S.E.M. for each group is also shown. P values were calculated using one-way ANOVA and Tukey multiple range test (A) and t-Test (B). ns, not significant. *p≤0.05, **p≤0.01, ***p≤0.001, ****p≤0.0001. The data underlying this figure can be found in S1 Data.
