# Supplementary material for: NAMPT-derived NAD+ fuels PARP1 to promote skin inflammation through parthanatos cell death
Source: PLoS Biol. 2021 Nov 8;19(11):e3001455. doi: 10.1371/journal.pbio.3001455 (PMC8601609; doi:10.1371/journal.pbio.3001455)
Supplement: S1 Table — (DOCX) [file pbio.3001455.s010.docx]

**Table S1.** Compounds used in this study.

| **Compound** | **Concentrations** | **Commercial branch** | **Catalog number** | **Target** |
| --- | --- | --- | --- | --- |
| FK-866 (Daporinad) | 1 µM | Selleckchem | S2799 | NAMPT |
|  | 10 µM |  |  |  |
|  | 100 µM |  |  |  |
| GMX1778 (CHS828) | 1 µM | Selleckchem | S8117 | NAMPT |
|  | 10 µM |  |  |  |
|  | 100 µM |  |  |  |
| Olaparib | 1 µM | Selleckchem | S1060 | PARPs |
|  | 10 µM |  |  |  |
|  | 100 µM |  |  |  |
|  | 250 µM |  |  |  |
|  | 500 µM |  |  |  |
|  | 1 mM |  |  |  |
| Veliparib | 1 µM | Sigma-Aldrich | S1004 | PARPs |
|  | 10 µM |  |  |  |
|  | 100 µM |  |  |  |
|  | 250 µM |  |  |  |
|  | 500 µM |  |  |  |
|  | 1 mM |  |  |  |
|  | 250 mM |  |  |  |
|  | 500 mM |  |  |  |
| Talazoparib | 100 nM | Sigma-Aldrich | S7048 | PARPs |
|  | 1 µM |  |  |  |
|  | 10 µM |  |  |  |
|  | 100 µM |  |  |  |
| Tempol | 100nM | Enzo Life Sciences | ALX-430-081-G001 | ROS |
|  | 1 µM |  |  |  |
|  | 10 µM |  |  |  |
|  | 100 µM |  |  |  |
|  | 1 mM |  |  |  |
| Mito-Tempo | 1 µM | Enzo Life Sciences | ALX-430-150-M005 | Mitochondrial ROS |
|  | 10 µM |  |  |  |
|  | 100 µM |  |  |  |
|  | 250 µM |  |  |  |
|  | 1 mM |  |  |  |
| N-Phenylmaleimide (NP) | 10nM | Santa Cruz Biotechnology | sc-250486 | AIFM1 translocation |
|  | 100nM |  |  |  |
|  | 1 µM |  |  |  |
|  | 10 µM |  |  |  |
|  | 100 µM |  |  |  |
|  | 1 mM |  |  |  |
| Apocynin | 1 µM | TOCRIS | 4663 | NADPH oxidases |
|  | 10 µM |  |  |  |
|  | 100 µM |  |  |  |
|  | 250 µM |  |  |  |
|  | 500 µM |  |  |  |
| N-Acetyl-cysteine (NAC) | 1 µM | Sigma-Aldrich | A9165 | ROS |
|  | 10 µM |  |  |  |
|  | 100 µM |  |  |  |
|  | 1 mM |  |  |  |
|  | 10 µM | Selleckchem |  |  |
|  | 100 µM |  |  |  |
| Nicotinamide (NAM) | 1mM | Sigma-Aldrich | N0636 | N/A |
|  | 100 µM |  |  |  |
| Nicotine mononucleotide (NMN) | 1mM | Sigma-Aldrich | N3501 | N/A |
|  | 100 µM |  |  |  |
| Nicotinamide adenine dinucleotide (NAD) | 1mM | Sigma-Aldrich | N3014 | N/A |
|  | 500 µM |  |  |  |
|  | 250 µM |  |  |  |
