# Supplementary material for: NAMPT-derived NAD+ fuels PARP1 to promote skin inflammation through parthanatos cell death
Source: PLoS Biol. 2021 Nov 8;19(11):e3001455. doi: 10.1371/journal.pbio.3001455 (PMC8601609; doi:10.1371/journal.pbio.3001455)
Supplement: Attachment — Submitted filename: 114802_1_rebuttal_2097400_q1qjt9.pdf [file pbio.3001455.s012.pdf]

Reviewer #1 (Evidence, reproducibility and clarity (Required)):

Summary:

In this study authors investigated the role of NAMPT, NAD+ and PARP1/parthanatos in skin inflammation using a zebrafish psoriasis model with an hypomorphic mutation of spint1a and human organotypic 3D skin models of psoriasis. Authors showed that genetic deletion and/or pharmacological inhibition of Nampt/PARP1/AIFM1/NADPH oxidases reduced oxidative stress, inflammation, keratinocyte DNA damage, hyperproliferation and cell death in zebrafish models of chronic skin inflammation. Authors also showed the expression of pathology-associated genes in human organotypic 3D skin models of psoriasis with pharmacological inhibition of Nampt/PARP1/AIFM1/NADPH oxidases. The key finding of this study is that PARP1 hyperactivation caused by ROS-induced DNA damage mediates skin inflammation through parthanatos.

Major comments:

This is a very comprehensive study to investigate the role of PARP1 in skin inflammation. The main conclusion was made based on the genetic inhibition and/or pharmacological inhibition of Nampt/PARP1/AIFM1/NADPH oxidases. Although the finding of this study that NAMPT-derived NAD+ fuels PARP1 to promote skin inflammation through parthanatos is interesting and important, there are lots of major concerns and questions, which have to be addressed to better support the main conclusion. In addition, the data and methods were not presented with sufficient detail.

1. This study is heavily relied on pharmacology inhibition. However, the specificity and selectivity of many inhibitors were not tested in this study.

At least 3 concentrations of each inhibitor were tested and the lowest one able to rescue the phenotype was then used for further testing (please, see Table S1). More importantly, the specificity of all compounds used were confirmed by genetic inhibition of their targets.

2. Fig. 1: it is quite confusing how NAD+ increases H2O2 levels? Is NAD+ cell permeable? It is not clear if NAD+ has been really up taken by cells in the larvae. If NAD+ fuels PARP1 to promote skin inflammation, why NAM treatment increased H2O2 levels but NMN precursor failed to increase skin oxidative stress? No reasonable explanation has been provided.

This is an interesting point. We have shown that exogenous NAD+ added in the water of larvae increased larval NAD+ (please, see Fig. 2K). It has been shown that neurons can take up NAD+ through CX43 (Fig. S7), so a similar mechanism may operate in larval skin. As regards, the effect of NAM and NMN, a recent study has demonstrated that NAM supplementation increased zebrafish larval NAD+; however, NA, NMN and NR failed to boost larval NAD+ level (PMID: 32197067). These results are consistent with our data.

3. Fig. 1E and 1G: it is not clear what is the green channel. Similarly, there is no clear description what is red or green in many other figures.

To help the interpretation of larval pictures, we have indicated in all figures what is analyzed in each fluorescent channel.

4. Fig. 1K and 1L: It is hard to understand why FK-866 reduced H2O2 release, but it increased neutrophils infiltration. How to interpret this conclusion?

5. Fig. 2C-D: Why low doses FK-866 reduced neutrophil infiltration whereas high dose FK-866 increased neutrophil infiltration?

Answer to 4&5: As it was explained in lines 145-156, FK-866 induces NF-kB activation in the muscle and neutrophil infiltration in this tissue when used at 100 uM. This result may be deleted if the reviewers think it is confusing, since a 10 uM dose was used in all subsequent experiments to study the impact of Nampt in skin inflammation. This dose has no effects in the muscle but robustly reduced skin H2O2 production and neutrophil skin infiltration.

6. Fig. 2I-J: it is not clear how NF-kB activity was measured. Is that based on green fluorescence shown in Fig. 2J? if so, the representative images were not consistent with the quantification data shown in I. Similarly, many other representative images were also not consistent with their quantification data throughout the manuscript. For example, Fig. 3C/D, 3E/F, 3G/H, 3L/M, Figure S2C/D, S2G/H, Fig. 4C/D, 4J/K.

The quantification of NFkB was measured in the skin, as it has already been reported previously (Candel et al., 2014). This is indicated in M&M section. The images show the whole larvae and NFkB is expressed at high levels in different tissues, such as neuromasts. To clarify this, we have included an additional figure to explain the ROI used for quantification of H2O2 and NfkB (Fig. S1G).

7. Figure S1C, Nampta/Namptb protein expression should be checked and shown after its KO using crispr/cas9 technique.

Unfortunately, we have used to different antibodies and both failed to crossreact with zebrafish Nampta/b. However, we have included the efficiency of CRISPR-Cas9 in Fig. S1F of the revised version. The efficiency is relatively low, probably indicating that is indispensable for zebrafish development, as occurs in mice (PMID 28333140).

8. Fig. 3I: protein expression of nox1, nox4 and nox 5 should be checked after genetic inhibition using CRISPR/Cas9 technique.

Unfortunately, we do not have antibodies able to recognize zebrafish Nox1, Nox4 and Nox5. However, we have provided the efficiency of the gRNA used for each gene (Fig. S3) and it is about 65%.

9. Fig. 4: If Olaparib treatment increased DNA damage, will it increase PARP1 activation and PAR formation?

As it has widely used in mammalian models, parthanatos is triggered by overactivation of PARP1 following DNA damage. Therefore, although inhibition of olaparib may further induces DNA damage, it blocks parthanatos. This is consistent with our results showing that olaparib reduces PARylation (Fig. S4H) and cell death (Figs. 4J, 4K).

10. Fig. 4M: it is not clear what staining has been done. No difference was observed among different groups.

As indicated in the figure legends, pγH2Ax^{+} (green) keratinocytes (red) are shown. We have indicated this in the figure and include arrows to show pγH2Ax^{+} cells. The quantitation of this

experiment (Fig. 4L) show that FK-866 robustly reduced, while olaparib increases, keratinocyte DNA damage.

11. Authors used N-phenylmaleimide (NP) to block AIF nuclear translocation. How does this inhibitor work? what is its actual effect on AIF nuclear translocation? Experiments are required to show this inhibitor actually blocks AIF nuclear translocation.

NP has been shown to block AIFM1 nuclear translocation, since it inhibits cysteine proteases which are required for its cleavage which precedes nuclear translocation (PMID 8879205). Although we have shown that genetic inhibition of Aifm1 rescues skin inflammation, confirming the specificity of the inhibitor, we agree on this point. Therefore, we have performed additional experiments and showed nuclear Aifm1 in keratinocyte aggregates of Spint1-deficient larvae and that NP treatment blocked nuclear translocation (Fig. S6C). In addition, we have also shown increased nuclear translocation of AIFM1 in keratinocytes of lesional skin from psoriasis patients (Figs. 6C, 6D).

12. Figure S4: it is hard to understand why lane #2 with Olaparib has the highest PAR signal.

We are sorry for this mistake labeling the WB. The right legend is: 1 +/+, 2 -/- treated with DMSO, 3 -/- treated with FK-866 and 4 -/- treated with olaparib.

13. Does spint1a-/- zebrafish show parthanatos cell death? It is not clear how cell death was measured.

We have shown that skin keratinocytes from Spint1a-deficient fish show increased cell death, as assayed by TUNEL, that is fully reversed by olaparib (Figs. 4J, 4K). In addition, skin keratinocytes from the mutant fish also have increased PARylation that is reversed by either FK-866 or olaparib (Fig. S4G, S4H). Further, pharmacological and genetic inhibition of Aifm1 inhibition and forced expression of Parga also rescue skin inflammation. Finally, we have included new experiments showing Aifm1 nuclear translocation in both Spint1a-deficient larvae and psoriasis patient lesional skin. Therefore, all these results show that Spint1a-deficient fish show parthanatos cell death-induced inflammation.

14. NAD+ levels were regulated by 3 different pathways. Expression of many genes involved in these 3 pathways were altered in psoriasis. However, it is not clear if the other two pathways play a role in PARP1-mediated inflammation.

NAD+ salvage pathway has been shown to be the major pathway regulating NAD+ levels in most tissues. The inhibition of this pathway with FK-866 rescues all skin phenotypes observed in Spint1a-deficient larvae as well as in organotypic 3D skin models of psoriasis. These results were further validated using another inhibitor (GMX1778) and genetic inhibition. Therefore, our results support that the salvage pathway is the one involved in psoriasis and inhibition of this pathway would rescue inflammation. However, it will be worthy to investigate if other pathways play a role in psoriasis and specifically upon inhibition of the salvage pathway.

Minor comments:

1. Page 9: To test this hypothesis, we used N-phenylmaleimide (NP), a chemical inhibitor of Aifm1 translocation from the nucleus to the mitochondria (Susin et al., 1996). The statement is not correct.

We are sorry for this mistake. It has been amended to: “To test this hypothesis, we used N-phenylmaleimide (NP), a chemical inhibitor of Aifm1 translocation from the mitochondria to the nucleus (Susin et al., 1996).”

2. Page 12: To the best of our knowledge, this is the first study demonstrating the existence of parthanatos in vivo. This statement is not correct.

We have removed this statement.

3. Figure S3 and S6E: they should be presented in an easy understandable way for the general readers.

We have explained in the legends the graph output of TIDE analysis.

4. Figure legends should be presented in a clearer way.

We have tried our best writing the legends. All suggestions and request were made.

Reviewer #1 (Significance (Required)):

Parthanatos is a new type of cell death distinct from apoptosis, necrosis, necroptosis and plays a pivotal role in ischemic stroke and neurodegenerative diseases (Wang Y et a., Science. 2016; Kam TI et al., Science 2018). The current study may provide new evidence of the importance of PARP1 and parthanatos in skin inflammation and potential targets for the treatment of skin inflammation.
We thank the reviewer’s opinion on the significance of our study.

The reviewer has the expertise in oxidative stress, PARP1 and parthanatos research.

Reviewer #2 (Evidence, reproducibility and clarity (Required)):

Summary:

The manuscript entitle "NAMPT-derived NAD+ fuels PARP1 to promote skin inflammation through parthanatos" is well written, divided and organized. This work demonstrated that models of psoriasis are characterized by ROS stress, inflammation and cell death. It was clear that NAMPT, a rate-limiting enzyme of NAD salvage pathway, and PARP1, a Poly-ADP-ribose polymerase, could be targeted to decrease ROS stress and inflammation that are contributing to cell death through parthanatos pathway. However, it was not clear that NAD+ are the responsible for fuel these processes in the psoriasis models analyzed. Nevertheless, the present work demonstrated that the cell death observed in the psoriasis model analyzed was correlated to an unidentified programmed cell death pathway, parthanatos that up to date has not been demonstrated.

We are pleased with the reviewer’s comments on our study.

Major comments:

Most of the data showed confirmed that inhibition of NAMPT or PARP1 seems to be beneficial for the relief of some characteristics related to oxidative stress and inflammation in the skin. However, the author should show data about NAD+ levels only instead of the ratio

NAD+/NADH to state that NAMPT-derived NAD+ is promoting oxidative stress (line 366-368) (fig2K).

The data shown in Fig 2K are NAD+ plus NADH. Considering that cytosolic and nuclear NAD+/NADH ratio typically ranges from 100 to 1000 (PMID: 21982715), these data mainly show intracellular NAD+ concentration in larvae.

Some data images are not convincing, or they don't really show an increase or decrease as the author showed in graph data. (Fig1D, 1E - 1F,1G).

The quantification of H_2O_2 and NFkB was measured in the skin, as it has already been reported previously (Candel et al., 2014). To clarify this, we have shown the ROI used for quantification of H_2O_2 and NfkB in Fig. S1G.

What is the relevance to analyze muscle and what is the relevance of the results obtained, since the effect of FK-866 in muscle increases the NFKB activity?

This is essentially a similar concern raised by reviewer 1. FK-866 induces NF-kB activation in the muscle and neutrophil infiltration in this tissue when used at 100 uM. This result may be deleted if the reviewers think it is confusing, since a 10 uM dose was used in all subsequent experiments to study the impact of Nampt in skin inflammation. This dose has no effects in the muscle but robustly reduced skin H_2O_2 production and neutrophil infiltration.

Figure S4H is not convincing with what the author wrote.

We are sorry for this mistake labeling the WB. The right legend is: 1 +/+, 2 -/- treated with DMSO, 3 -/- treated with FK-866 and 4 -/- treated with olaparib. Both FK-866 and olaparib rescue PARylation in the skin of Spint1a-deficient larvae.

The author should make the keratinocyte aggregation experiment with FK-866 treatment to better substantiate what they are proposing.

These results are shown in Figs. 2E and 2F.

Minor comments:

Line 281: "NP, a chemical inhibitor of Aifm1 translocation from the nucleus to the mitochondria..." should be the opposite: NP, a chemical inhibitor of Aifm1 translocation from mitochondria to nucleus.

We are sorry for this mistake. It has been amended.

Line 299 "figure 6A" should be Figure 6B.

We have checked and it is correct.

How the author explains the relationship between all the results being related to NAMPT and supposedly to NAD+, but an important precursor to make NAD through salvage pathway (NMN) and a well NAD+ booster didn't show any effect?

This is an interesting point that was also raised by reviewer 1. A recent study has demonstrated that NAM supplementation increased zebrafish larval NAD+; however, NA, NMN and NR failed to boost larval NAD+ level (PMID: 32197067). This explains our results. We have discussed this point in the revised manuscript.

Line 178: should be NAMPT inhibitor stead of FK-866 inhibitor.

Thanks a lot. It has been amended.

Line 191-192: I suggest reformulating this sentence since the result showed was only the ratio NAD/NADH.

Please, see our response above. We are measuring NAD+ plus NADH. We have amended the text to clarify this fact.

Reviewer #2 (Significance (Required)):

The present work greatly demonstrated the relevance of PARP1 and NAMPT in the field of inflammation and ROS in the skin that contribute to diseases like psoriasis. Although it is not a lethal disease, as the author mentioned, it affects the physical and mental health of the individual. Understanding the mechanism that underlie this condition would help to trigger new and more efficient treatments. It was clear that the result showed a promising strategy in targeting NAMPT and PARP1. Furthermore, inhibitor for them is already know and may be useful for future treatment of psoriasis disease.
We thank this comments on the impact of our study.

Reviewer #3 (Evidence, reproducibility and clarity (Required)):

This study shows NAMPT derived NAD facilitates PARP activation to promote skin inflammation via parthanatos. The authors used the zebrafish model and organoid models of psoriasis and observed that inhibition of NAMPT reduces inflammation in zebrafish and human skin organoid models. They also observed that NADPH oxidase-derived oxidative stress activates PARP, and PARP inhibition or over-expression of PARG or AIF mimics protection mediated by NAMPT inhibition. This is an interesting study, but there are several weaknesses to support the conclusions of this study. While pharmacological inhibition is a powerful tool, complementary methods (knock out of PARP-1) are critical for this paper's conclusions. PARP inhibitor used in this study may not specifically inhibit PARP1 but other PARPs too. Therefore, genetic knockout of PARP will make the make this conclusions/interpretation of this study strong.

We thank these comments on our manuscript. All pharmacological inhibitions used in this study were confirmed by genetic experiments, including Parp1. The genetic inhibition of Parp1 is shown in Figs. S4C-S4F.

Additional comments include:

This study's primary focus is PARP activation and PAR-mediated parthanatos, but it is not shown how different inhibitors used in this study and supplementations of NAD alter PARP activation and PAR formation.

We have shown through the quantitation of PARylation that Spint1a-deficient skin shows increased PAR activity and that pharmacological inhibition of either Nampt or Parp was able to fully reverse it (Figs S4g & S4H). In addition, we have also shown a dramatically increased PAR activity in lesional skin biopsies from psoriasis patients (Fig. 6E).

NAMPT is not the only NAD biosynthesis pathway; how other NAD pathways respond when NAMPT is inhibited with FK-866.

NAD+ salvage pathway has been shown to be the major pathway regulating NAD+ levels in most tissues. The inhibition of this pathway with FK-866 rescues all skin phenotypes observed in Spint1a-deficient larvae as well as in organotypic 3D skin models of psoriasis. Therefore, our results support that the salvage pathway is the one involved in psoriasis and inhibition of this pathway would rescue inflammation. However, we agree that it will be worthy to investigate if other pathways play a role in psoriasis and specifically upon inhibition of the salvage pathway. However, this is out of the scope of this manuscript.

PARG is used in this study, but the protein levels of PARG are not shown, and it is not clear whether the PARG overexpression is sufficient to reduce PAR levels in the models used.
AIF pharmacological and genetic manipulation of AIF is used, but it is not shown that AIF translocates to the nucleus in this model.

We agree on these points, so we have analyzed Aifm1 translocation in Spint1a-deficiet larvae and psoriasis patient lesional skin (please, see above our response to reviewer 1) and PARylation upon forced expression of Parga (Fig. 5M).

Does NAMPT inhibition reduce NAPD oxidase activity?

Our results indicate that Nampt inhibition reduce NAPDH oxidase activity, since a drastic reduction of H2O2 production was observed in the skin of Spint1a-deficient larvae treated with FK-866.

PAR plots provided in fig S4 need quantification, and the blots (Fig S4 G&H) should be run on the same gel to make sure the exposure levels are the same. It is not clear which group is represented in lane 4 of Fig S4 G.

We have provided the quantitation. The problem is that we mislabeled the legend of Fig. S4H. The right legend is: 1 +/+, 2 -/- treated with DMSO, 3 -/- treated with FK-866 and 4 -/- treated with olaparib. Therefore, either Nampt or Parp inhibition robustly reduces PARylation of Spint1a-deficient skin to the levels of their wild type counterparts.

Reviewer #3 (Significance (Required)):

This study in interesting potentially showing the role of PARP-1 activation and Parthanatos in skin inflammation. It could be very significant if above identified weaknesses are addressed.

We are pleased with this reviewer's assessment on the significance of our study.
