# Supplementary material for: NAMPT-derived NAD+ fuels PARP1 to promote skin inflammation through parthanatos cell death
Source: PLoS Biol. 2021 Nov 8;19(11):e3001455. doi: 10.1371/journal.pbio.3001455 (PMC8601609; doi:10.1371/journal.pbio.3001455)
Supplement: Attachment — Submitted filename: point by point reply-R2.pdf [file pbio.3001455.s014.pdf]

Reviewer 1:

In this revision, although authors addressed few questions, the majority of questions were not fully addressed. Please see details in blue (after authors' response) as following:
We are sorry but, respectfully, we disagree with you.

2. Fig. 1: it is quite confusing how NAD+ increases H2O2 levels? Is NAD+ cell permeable? It is not clear if NAD+ has been really up taken by cells in the larvae. If NAD+ fuels PARP1 to promote skin inflammation, why NAM treatment increased H2O2 levels but NMN precursor failed to increase skin oxidative stress? No reasonable explanation has been provided.

This is an interesting point. We have shown that exogenous NAD+ added in the water of larvae increased larval NAD+ (please, see Fig. 2K). It has been shown that neurons can take up NAD+ through CX43 (Fig. S7), so a similar mechanism may operate in larval skin. As regards, the effect of NAM and NMN, a recent study has demonstrated that NAM supplementation increased zebrafish larval NAD+; however, NA, NMN and NR failed to boost larval NAD+ level (PMID: 32197067). These results are consistent with our data.

Authors did not provide strong evidence that NAD+ is cell permeable. In contrast, lots of previous literatures showed that NAD+ is not cell permeable (see review Covarrubias AJ et al., Nature Reviews, 2021). It is still not clear how NAD+ increased H2O2. Authors did not address this question at all.

There are several papers demonstrating that zebrafish larvae are able to take up NAD+ and NADH (for example PMID 31609202 and 31391079), including a paper published in Plos Biology (PMID 23109907). In addition, several studies have also shown that NAD+ is taken up by different cells and tissues through connexin 43, for example hypothlamus (PMID 30179604) and fibroblasts (PMID 11099492). We have included these references in our revision version (lines 144-148).

We have already discussed how NAD+ increase oxidative stress in the discussion: “the mechanism involved in the In the models used, the ability of NAD+ and its precursors to induce oxidative stress can be explained by their capacity to boost NADH/NADPH intracellular levels that would fuel NADPH oxidases to generate ROS (Figure 8). In fact, pharmacological and genetic inhibition of NADPH oxidases or NAMPT efficiently counteracted skin H2O2 synthesis and inflammation in both zebrafish and human models.”

3. Fig. 1E and 1G: it is not clear what is the green channel. Similarly, there is no clear description what is red or green in many other figures.
To help the interpretation of larval pictures, we have indicated in all figures what is analyzed in each fluorescent channel.

Authors still did not fully address the question for all figures. For example, authors only showed that "Representative merge images (green and red channel) of lyz:dsRED zebrafish larvae of every group are shown (E, G)." in the figure legend, which does not clearly tell what is red and green.

We included in our first revision a figure (Fig. S1G) showing how neutrophil infiltration skin oxidative stress and NFkB activation was analyzed. Red neutrophils are clearly observed in all images and it is not possible to label the 150 neutrophils of 3 dpf larvae. As regards, H2O2 and NFkB, as these are reporter, are observed in whole larvae, so it is not possible to labelled them. Fig. S1G shows the ROIs placed in skin used for quantitation.

4. Fig. 1K and 1L: It is hard to understand why FK-866 reduced H2O2 release, but it increased neutrophils infiltration. How to interpret this conclusion?

Authors still did not address this question at all. This question is different from question 5.

As we have already explained in the first round of revision, the high dose of FK866 used (100 uM) induced NFKB activation in the muscle, not in the skin. This dose was not used further used but we decided to leave it in just Fig. 1, which shown the results of wild type fish not Spint1a-deficient, because it can be useful for other researchers working in muscle metabolism and NAD+.

6. Fig. 2I-J: it is not clear how NF-kB activity was measured. Is that based on green fluorescence shown in Fig. 2J? if so, the representative images were not consistent with the quantification data shown in I. Similarly, many other representative images were also not consistent with their quantification data throughout the manuscript. For example, Fig. 3C/D, 3E/F, 3G/H, 3L/M, Figure S2C/D, S2G/H, Fig. 4C/D, 4J/K.
The quantification of NFkB was measured in the skin, as it has already been reported previously (Candel et al., 2014). This is indicated in M&M section. The images show the whole larvae and NFkB is expressed at high levels in different tissues, such as neuromasts. To clarify this, we have included an additional figure to explain the ROI used for quantification of H2O2 and NfkB (Fig. S1G).

It is still unclear how NF-kB activity as measured. It was also not really indicated in M&M sections as authors mentioned. Authors also did not address the concerns that the representative images were not consistent with the quantification data shown in Fig. 3C/D, 3E/F, 3H/I, 3L/M, Figure S2C/D, S2G/H, Fig. 4C/D, 4J/K.

Please, refer to Fig. S1G because it is explained. All figures used were carefully checked and are representative. In fact, reviewer 1 and 3 agree with us and have no concerns about them.

7. Figure S1C, Nampta/Namptb protein expression should be checked and shown after its KO using crispr/cas9 technique.
Unfortunately, we have used to different antibodies and both failed to crossreact with zebrafish Nampta/b. However, we have included the efficiency of CRISPR-Cas9 in Fig. S1F of the revised version. The efficiency is relatively low, probably indicating that is indispensable for zebrafish development, as occurs in mice (PMID 28333140).

Fig. S1F was not presented clearly and was not explained in an easily understandable way.

We have already explained in the figures the output of a TIDE analysis: it only shows the INDELS at the target site and the specific efficiency of the gRNA used.

8. Fig. 3I: protein expression of nox1, nox4 and nox 5 should be checked after genetic inhibition using CRISPR/Cas9 technique.
Unfortunately, we do not have antibodies able to recognize zebrafish Nox1, Nox4 and Nox5. However, we have provided the efficiency of the gRNA used for each gene (Fig. S3) and it is about 65%.

Fig. S3 was not presented clearly and was not explained in an easily understandable way. It is difficulty to charge the efficiency of gRNAs based on these data. Checking their protein levels is

mandatory to see if the experimental conditions are successful.

The efficiency of the editing is >63% for all gRNA used (please, see our answer to point 7 for interpretation of TIDE output) and robust phenotype was obtained, which is consistent with the pharmacological inhibition of NADPH and ROS scavenging. Although it is not possible to confirm the knockdown by WB as there are no antibodies available, we think this is not required considering the high editing efficiency and the consistency of phenotypes observed.

9. Fig. 4: If Olaparib treatment increased DNA damage, will it increase PARP1 activation and PAR formation?
As it has widely used in mammalian models, parthanatos is triggered by overactivation of PARP1 following DNA damage. Therefore, although inhibition of olaparib may further induces DNA damage, it blocks parthanatos. This is consistent with our results showing that Olaparib reduces PARylation (Fig. S4H) and cell death (Figs. 4J, 4K).

No convincing evidence was provided that olaparib increased DNA damage in this manuscript. In contrast, authors showed that Olaparib reduced PARylation and cell death. PARP-1 is a DNA damage sensor and its biological functions are highly context dependent. In cancer research (without severe DNA damage), PARP-1 may contribute to DNA repair and PARP inhibitor may increase DNA damage leading to cancer cell death. However, following severe DNA damage, as authors mentioned that PARP-1 hyperactivation will promote parthanatos. It is puzzling how Olaparib treatment increased DNA damage but reduced PARylation. This conclusion sounds self-contradictory.

We are sorry but we disagree on this point. We have provided robust evidence that olaparib increase DNA damage by using 2 different techniques: pgH2AX WIHC of whole larvae and by comet assay of keratinocyte suspensions (Figs. 4L-4N). We agree, however, that the functions of olaparib are highly context dependent. Thus, we show in this study that DNA damage promote hyperactivation of Parp1 which then results in parthanatos. The reduction of DNA damage by olaparib and the reduced parylation are not contradictory: inhibition of Parp1 enzymatic activity results in increased DNA damage, particularly in Spint1a-deficient larvae as their keratinocytes are producing ROS at very high levels, and at the same time blocks parthanatos, which also depend on enzymatic activity of Parp1. This is extensively discussed in our manuscript and is the mechanisms already shown in several cell types in previous in vitro studies.

10. Fig. 4M: it is not clear what staining has been done. No difference was observed among different groups.
As indicated in the figure legends, pγH2Ax+ (green) keratinocytes (red) are shown. We have indicated this in the figure and include arrows to show pγH2Ax+ cells. The quantitation of this experiment (Fig. 4L) show that FK-866 robustly reduced, while olaparib increases, keratinocyte DNA damage.

The image quality of Fig. 4M is poor. Fig. 4M did not show any difference among different groups. It is quite surprising that Fig. 4N shows statistic significance among groups with such small difference. Again, if Olaparib increased DNA damage, how it reduced PARylation? PARP-1 is the DNA damage sensor and PARylation is the bioproduct of DNA damage and PARP-1 activation.

We think we have already addressed this concern, as reviewers 1 and 3 also agree. We appreciate that you are surprised about the statistical significance of the results shown in Fig.

4N but this is a subjective appreciation. Our results are consistent and the differences robust and statistically significant (100 cells in each condition were analyzed in the comet assay).

Minor
3. Figure S3 and S6E: they should be presented in an easy understandable way for the general readers.
We have explained in the legends the graph output of TIDE analysis.

Authors still did not present Fig. 1F, S3, S4F and 6F clearly and did not explain the data in an easily understandable way.

Please, see our response to point 7.

Reviewer 2:
The authors have addressed all my comments, and also the comments of the other reviewers. I believe that the manuscript is quite interesting and has potential clinical relevance.

We are pleased with the reviewer's comments and having addressed all concerns.

Reviewer 3:
The revised manuscript has been substantially improved. However, some weaknesses that need to be addressed include:
1. The AIF pictures are very poor, better quality pictures with data needs quantification is required. In addition, cell fractionation experiments for levels of AIF in nuclear and mitochondrial fractions are critical for the interpretation of these results.
We have provided high resolution images (tif) but taking these images is quite challenging, since we are imaging nuclear translocation of Aifm1 in whole zebrafish larvae. Cellular fractionation followed by western blot is not sensitive enough to detect Aifm1 translocation in our experimental setup, since just a few keratinocytes in the whole larvae showed nuclear Aifm1, probably because these cells quickly die and are rapidly cleared by immune cells. This is consistent with our TUNEL assay where just a few positive cells are also observed, and an earlier study reporting the phenotype of Spint1a-deficient larvae (Carney et al., 2007).

2. While PARG mRNA has been used in the revisions but no data is provided that the PARG levels indeed increase in the cells/larvae.
Although we have shown that *parga* injection robustly reduces parylation in Spint1a-deficient larvae (Fig. 5M), which demonstrates increased Parga activity in injected embryos, we have now included in the revised version *parga* mRNAs levels, too (Fig. 5N).

3. NAD quantification following NAMPT modulation vs PARP inhibition has not been shown.
Overall, this is an interesting study but the above mentioned concerns need to be addressed to make the interpretations/conclusions of this study clear.
We have determined NAD+ content in larvae treated with NAD+, FK-866 and olaparib by an enzymatic method. The new data confirm the results obtained by ELISA for NAD+ and FK-866 treatments and show that olaparib fails to alter larval NAD+ content. The results are shown in Fig. 2L of the revised version.
